# Echocardiographic factors associated with prolonged duration of inotrope therapy and ICU length of stay in a retrospective study of cardiac surgery patients

**DOI:** 10.62838/jccm-2026-0013

**Published:** 2026-04-30

**Authors:** Kelly Tankard, William Vasquez McTeigue, Matthew Smith, Ariel Mueller, Timothy Houle, Eriberto Michel, Judy Hung, Adam Dalia, Jerome Crowley, Kenneth Shelton

**Affiliations:** Department of Anesthesiology, University of Kansas Medical Center, Kansas City, USA; Department of Anesthesiology, Massachusetts General Hospital, Boston, USA

**Keywords:** critical care, cardiac surgery, echocardiography, heart failure, inotrope

## Abstract

**Introduction:**

Post-operative heart failure following cardiac surgery carries risk and can impact patient outcomes. Preoperative echocardiography can be useful for stratifying risk. Although there has been a historical focus on left ventricular ejection fraction (LVEF), the importance of left ventricular (LV) size, as measured by left ventricle end-diastolic diameter (LVEDD), may be an underappreciated echocardiographic factor which can help predict risk in patients undergoing cardiac surgery.

**Aim of the study:**

To investigate the association between LVEF and LVEDD with inotrope use, inotrope duration, and intensive care unit (ICU) length of stay (LOS) in patients undergoing cardiac surgery.

**Materials and methods:**

Retrospective cohort study including 2,965 adult patients undergoing non-emergent cardiac surgery at a single academic institution between February 2017 and October 2021. Primary outcomes were the use of inotropes and duration of inotrope therapy. The secondary outcome was ICU LOS.

**Results:**

In adjusted analyses, a one standard deviation increase in LVEF was associated with decreased odds of inotrope initiation (OR 0.45, 95% CI: 0.41 to 0.50; P < 0.001), while a one standard deviation increase in LVEDD was associated with increased odds of receiving inotropes (OR 1.18, 95% CI: 1.07 to 1.31; P = 0.001). Among those receiving inotropes, a one standard deviation increase in LVEF was associated with a 25% decrease in inotrope hours in adjusted analyses (0.75, 95% CI: 0.68 to 0.82; P < 0.001). An interaction was observed such that LVEDD modified the association between LVEF and ICU LOS (0.98, 95% CI: 0.95 to 0.99; P = 0.03).

**Conclusions:**

Preoperative LVEDD, particularly when combined with LVEF, can predict risk after cardiac surgery.

## Introduction

Heart failure, or more specifically, low cardiac output syndrome (LCOS), is estimated to affect up to 10% of all post-cardiac surgical patients each year, leading to increased mortality and postoperative complications in the intensive care unit [1]. Inotrope therapy is routinely used perioperatively in cardiac surgery to improve cardiac output in these patients. A cardiac index (CI) below 2.0 L/min/m^2^, a systolic blood pressure below 90 mmHg, and the presence of tissue hypoperfusion may indicate LCOS [2, 3]. Patients requiring prolonged inotrope therapy suffer adverse patient outcomes, including increased mortality and prolonged intensive care unit (ICU) length of stay (LOS) [4].

Echocardiography is helpful in identifying patients at risk for postoperative heart failure who may require inotropes. Left ventricular ejection fraction (LVEF) is routinely measured and reported by preoperative transthoracic echocardiography and often used to identify patients at risk. Prior studies have assessed LVEF, its association with ease of separation from cardiopulmonary bypass (CBP), and the need for inotrope therapy [5,6,7,8]. Prolonged cardiovascular pharmacological support has been associated with an LVEF < 30% [9]. Patients with low pre-operative LVEF undergoing cardiac surgery are also at higher risk of post-operative complications [10].

Although there is a historical focus on LVEF and its relation to outcomes after cardiac surgery, there is limited data on the effect of left ventricular (LV) size, as measured by left ventricular end-diastolic diameter (LVEDD), on cardiac surgical outcomes. Changes in LVEDD can be a marker of chronic heart failure, and although echocardiographic laboratories routinely report LVEDD, it is rarely mentioned as a potential risk factor for poor perioperative outcomes. While it may be intuitive that patients with abnormal LV size due to chronic heart failure are at higher risk, there is little data to support this. Additionally, research on the combination of LVEDD and LVEF to better predict inotrope need after cardiac surgery is lacking. In addition to inotrope usage, ICU length of stay is an important hospital quality metric, and prolonged ICU LOS is associated with adverse postoperative outcomes and increased hospital costs [11]. Prolonged ICU LOS has previously been associated with low LVEF; however, LVEDD has yet to be included in prior studies [12]. Our study aimed to focus on LV size and its relation to LVEF on outcomes after cardiac surgery.

Echocardiographic variables may be associated with the initiation and duration of inotrope therapy, along with ICU LOS, and therefore may serve as powerful predictors of perioperative risk. In this study, we hypothesized that two echocardiographic indices – LVEF and LVEDD – would be independently associated with changes in inotrope use, inotrope duration, and ICU LOS.

## Materials and Methods

### Study Design

This retrospective cohort study examined the relationship between LVEDD, LVEF, hours of inotrope therapy, and ICU LOS in cardiac surgery patients. The study was conducted at Massachusetts General Hospital (MGH) with patients 18 years and older. Adult patients who underwent cardiac surgery between February 1^st^, 2017, and October 31^st^, 2021, were included. Patients with missing lengths of stay or echocardiographic indices (LVEDD or LVEF), emergent surgeries, patients on mechanical circulatory support at any point during their hospital stay, and echocardiograms conducted more than a year before surgery were excluded from the analysis, as were patients who died during the hospital admission. This study was approved by the Institutional Review Board with a waiver of informed consent (Protocol #2021P000665).

### Data Collection

Data was collected from the MGH Echocardiography Laboratory Database, the Society of Thoracic Surgeons (STS) database, and the electronic medical record. Preoperative transthoracic echocardiogram (TTE) measurements of LVEF and LVEDD were abstracted from the Echocardiography Laboratory Database. Patient characteristics and procedural variables such as age, sex, hypertension, diabetes, cardiopulmonary bypass time, presence of a tracheostomy, and ICU LOS were abstracted from the STS database. The LVEDD from STS supplemented any missing values of LV size from the Echocardiography Laboratory. The American Society of Anesthesiology (ASA) score, chronic kidney disease, acute renal replacement therapy, revision sternotomy, and hours of inotrope therapy were abstracted from the electronic medical record via structured queries.

Pre-operative TTE data was used for determination of baseline echocardiographic data, as the day of surgery, intra-operative transesophageal echocardiography (TEE) reports at our institution typically do not report quantitative measures of LVEF nor LVEDD. Furthermore, there may be hemodynamic changes under anesthesia (nil per os status, lower afterload due to anesthetics, withholding of goal-directed medical therapies prior to surgery), which would make intra-operative echocardiography measurements less relevant than pre-operative TTE. We used data from the most recent TTE prior to surgery, excluding patients from our study who did not have a TTE within one year of surgery. At our institution, pre-operative TTEs are ordered within one year of surgical date or with any significant clinical change prior to surgery, and therefore our TTE data was presumed to best represent the baseline echocardiographic characteristics of the patients.

The primary outcome evaluated the presence of inotrope use and its duration in hours. Inotrope usage was defined as administration of an infusion of any of the following medications or any combination: epinephrine, dobutamine, milrinone, and dopamine. The total duration of inotrope therapy was calculated by summing the infusion times that an inotrope was being administered in the ICU. ICU LOS was evaluated as a secondary outcome, with duration reported in hours.

### Statistical Analysis

Descriptive data were reported as median (interquartile range [IQR]) or frequency (percentage) depending on the variable type. Absolute standardized differences were calculated to assess the balance between patients with and without inotrope use. Spearman's correlation coefficient was calculated between preoperative LVEF and LVEDD. The primary outcome, the number of inotrope hours, and its association with LVEDD and LVEF were investigated via a two-layer hurdle model. The presence of inotrope administration was first examined using logistic regression. Results are reported as odds ratio (OR), 95% confidence interval (CI), and its associated p-value. In the second step of the hurdle model, the association between echocardiographic indices and the duration of inotrope administration was evaluated among those who received inotropes. Given the skewed distribution observed upon visual inspection of the data, the number of inotrope hours was log-transformed and investigated with linear regression. As a result, coefficient estimates were exponentiated, and the results were reported as percent changes, 95% CI, and p-values. Both aspects of the hurdle model were initially performed crudely and then were adjusted with the following prespecified covariates, namely American Society of Anesthesiologists (ASA) physical status, age (centered and scaled), sex, hypertension, diabetes, chronic kidney disease, cardiopulmonary bypass time in minutes (centered and scaled), and revision sternotomy. Covariates were selected a-priori based on their associations with the outcomes. The final inference was made using the fully adjusted models.

The secondary outcome, ICU LOS, and its association with LVEDD and LVEF were similarly assessed with log-transformations and linear regression models. Unadjusted and adjusted models were constructed using the same covariates specified above, with the addition of acute renal replacement therapy and presence of a tracheostomy. In a final exploratory analysis an interaction term was included between the LVEDD and LVEF. Coefficient estimates were also similarly exponentiated, and results were reported as percent changes, 95% CI, and p-value. In a sensitivity analysis for both the primary and secondary outcome, each adjusted model was subsequently controlled for body surface area. In each model formulation, LVEDD and LVEF were centered and scaled, with effect estimates thus reported for a one standard deviation increase in the echocardiographic index. No a priori power analysis was performed. Instead, all available data during the study period were included. All analyses were performed using R (4.4.0) and RStudio (2024.04.0) with two-sided p-values < 0.05 considered statistically significant.

## Results

**Fig. 1.**
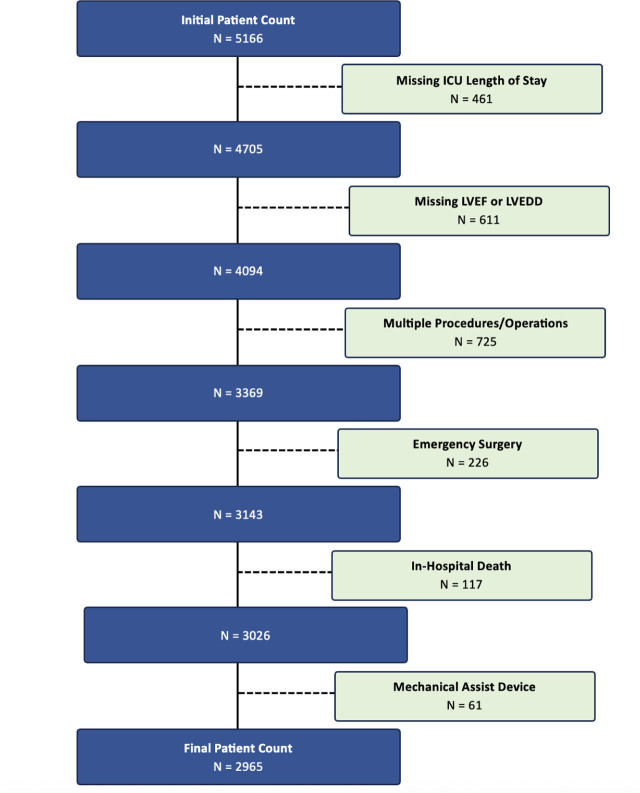
**Study Cohort.** Details of the inclusion and exclusion criteria are outlined at each step while assembling the study cohort. In total 2,965 patients were included in the final analysis.

Data was extracted on a total of 5,166 patients. Of these, 461 were excluded due to missing ICU hours and 611 were excluded due to missing LVEF or LVEDD measurements. An additional 725 patients were excluded to remove patients who had subsequent and separate surgical procedures under a different hospital admission during the study period. Additional exclusions included patients of emergent status (n = 226), hospital deaths (n = 117), and those receiving mechanical assist devices at any point during their hospitalization (n = 61; Figure 1). After the exclusions described above, a total of 2,965 ICU patients were eligible and included in the final analysis.

Of the included participants, the median age was 67 (IQR 59, 74) years, with a higher proportion of males (73%) in the cohort (Table 1). The majority of included participants reported an ASA physical status of 3 (54%) or 4 (44%). The median preoperative LVEF was 62% [IQR 55%, 67%] among all participants and median LVEDD was 49 [IQR 44, 54] millimeters. Preoperative LVEF was negatively associated with LVEDD (Spearman's ρ = −0.36). Inotrope therapy occurred in 992 (33%) patients, with a median duration of inotrope administration of 23 [IQR 11, 55] hours among those receiving this therapy.

**Table 1.** Patient baseline, procedural, and encounter characteristics

	**Overall N = 2965**	**Absence of Inotropes N = 1973**	**Presence of Inotropes N = 992**	**ASD**
Age, years	67 [59, 74]	66 [58, 73]	69 [61, 75]	0.25
Male	2166 (73)	1499 (76)	667 (67)	0.20

ASA Classification				0.36
2	56 (2)	47 (2)	9 (1)	
3	1591 (54)	1163 (60)	428 (43)	
4	1308 (44)	758 (38)	550 (55)	
5	4 (0)	2 (0)	2 (0)	
Missing	6 (0)	3 (0)	3 (0)	

Diabetes	881 (30)	523 (27)	358 (36)	0.21
Hypertension	2386 (81)	1554 (79)	832 (84)	0.13
Chronic Kidney Disease	401 (14)	207 (11)	194 (20)	0.26
Acute Renal Replacement Therapy	54 (2)	10 (1)	44 (4)	0.26
Tracheostomy	40 (1)	6 (0)	34 (3)	0.23
CABG	1602 (54)	1051 (53)	551 (56)	0.05
Aortic valve procedure	1078 (36)	752 (38)	326 (33)	0.11
Mitral valve procedure	763 (26)	382 (19)	381 (38)	0.43
Tricuspid valve procedure	160 (5)	41 (2)	119 (12)	0.40
Pulmonic valve procedure	6 (0)	3 (0)	3 (0)	0.03
CABG and valve procedure	467 (16)	252 (13)	215 (22)	0.24
Thoracic aortic procedure	434 (15)	297 (15)	137 (14)	0.04
Other cardiac procedure	650 (22)	352 (18)	298 (30)	0.29
Redo Sternotomy	9 (0)	5 (0)	4 (0)	0.03
Cardiopulmonary Bypass Time, minutes	131 [97, 169]	121 [93, 154]	155 [114, 195]	0.58

LVEF	62 [55, 67]	63 [58, 68]	58 [43, 65]	0.66
Normal, >= 50%	2490 (84)	1822 (92)	668 (67)	
Low, <50%	475 (16)	151 (8)	324 (33)	

LVEDD	49 [44, 54]	48 [43, 53]	51 [45, 56]	0.30
Dilated^a^	460 (15)	236 (12)	224 (23)	
Normal^a^	2220 (75)	1523 (77)	697 (70)	
Small^a^	285 (10)	214 (11)	71 (7)	

30 Day Morality	10 (0)	3 (0)	7 (1)	0.09
ICU Length of Stay, hours	28 [23, 53]	25 [22, 40]	55 [30, 110]	0.55

Data is presented as median [quartile 1, quartile 3] and frequency (percentage) depending on variable type. Abbreviations: Absolute standardized difference (ASD), American Society of Anesthesiology (ASA), Coronary artery bypass grafting (CABG), left ventricle ejection fraction (LVEF), left ventricle end-diastolic diameter (LVEDD), Intensive Care Unit (ICU). aLVEDD is categorized as follows: Small (male: < 42; female: < 37), Normal (male: 42 to 58; female: 37 to 52), Dilated (male: > 58; female: > 52)

Results from unadjusted and adjusted models looking at the presence of inotrope administration in relation to LVEDD and LVEF are shown in Table 2. A one standard deviation increase in LVEF was associated with a decrease in the odds of administering inotrope therapy (unadjusted OR 0.50, 95% CI: 0.46 to 0.54; P < 0.001 and adjusted OR 0.45, 95% CI: 0.41 to 0.50; P < 0.001). Conversely, a one standard deviation increase in LVEDD was associated with an increase in the odds of administering inotrope therapy (unadjusted OR 1.36, 95% CI: 1.26 to 1.48; P < 0.001 and adjusted OR 1.18, 95% CI: 1.07 to 1.31; P = 0.001).

**Table 2.** Association of LVEF and LVEDD with the presence of ICU inotrope therapy

	**Odds Ratio (95% CI)**	**P Value**
LVEF, unadjusted	0.50 (0.46 to 0.54)	<0.001
LVEDD, unadjusted	1.36 (1.26 to 1.47)	<0.001
LVEF, adjusted	0.45 (0.41 to 0.50)	<0.001
LVEDD, adjusted	1.18 (1.07 to 1.31)	0.001

Adjusted models include the following prespecified variables: age (centered and scaled), sex, ASA, presence of diabetes, presence of hypertension, presence of chronic kidney disease, cardiopulmonary bypass time (centered and scaled), and presence of redo sternotomy. As LVEF and LVEDD have been centered and scaled, odds ratio are interpreted per unit standard deviation. Abbreviations: left ventricle ejection fraction (LVEF), left ventricle end-diastolic diameter (LVEDD).

When only investigating patients with inotrope administration, a one standard deviation increase of LVEF was associated with 25% decrease in the duration of inotrope therapy (adjusted 0.75, 95% CI: 0.68 to 0.82; P < 0.001; Table 2). No evidence of an association was observed between LVEDD and duration of inotrope therapy in either unadjusted or adjusted analyses.

**Fig. 2.**
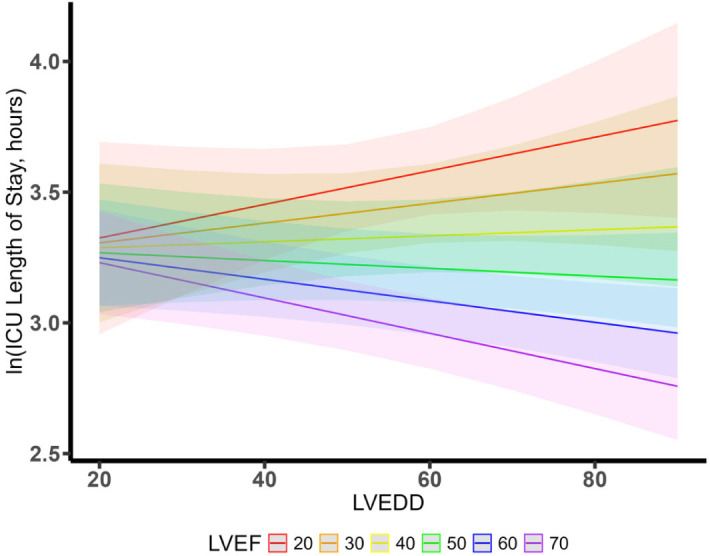
**Interaction Between LVEF and LVEDD on ICU Length of Stay.** The association between left ventricular end-diastolic diameter (LVEDD) and the natural log (ln) of ICU length of stay is reported for varying categories of left ventricular ejection

When assessing ICU LOS as a secondary outcome, a significant interaction was observed between LVEF and LVEDD (adjusted 0.98. 95% CI: 0.95 to 0.99; P = 0.03; Table 3). As shown in Figure 2, LVEF modified the association between LVEDD and ICU LOS, in which patients with low LVEF and dilated LVEDD resulted in longer ICU LOS, whereas among those with preserved LVEF, a dilated LVEDD was associated with a reduction in ICU LOS.

**Table 3.** Association of LVEF and LVEDD with hours of ICU inotrope therapy

	**Effect Estimate (95% CI)**	**P Value**
LVEF, unadjusted	0.88 (0.81 to 0.96)	0.003
LVEDD, unadjusted	0.97 (0.89 to 1.05)	0.41
LVEF, adjusted	0.75 (0.68 to 0.82)	<0.001
LVEDD, adjusted	0.96 (0.88 to 1.06)	0.45

Only patients with ICU inotrope administrations were included. Adjusted models include the following prespecified variables: age (centered and scaled), sex, ASA, presence of diabetes, presence of hypertension, presence of chronic kidney disease, cardiopulmonary bypass time (centered and scaled), and presence of redo sternotomy. LVEF and LVEDD were centered and scaled. Effect estimates have been exponentiated and are interpreted as percent changes per unit standard deviation. Abbreviations: left ventricle ejection fraction (LVEF), left ventricle end-diastolic diameter (LVEDD).

**Table 4.** Association of LVEF and LVEDD with ICU hours

	**Effect Estimate (95% CI)**	**P Value**
Multivariable Interaction		
LVEF, unadjusted	0.88 (0.86 to 0.91)	<0.001
LVEDD, unadjusted	0.94 (0.92 to 0.97)	<0.001
LVEF * LVEDD, unadjusted	0.97 (0.94 to 0.99)	0.01

Multivariable Interaction		
LVEF, adjusted	0.89 (0.87 to 0.92)	<0.001
LVEDD, adjusted	0.97 (0.94 to 0.99)	0.02
LVEF * LV, adjusted*	0.98 (0.95 to 0.99)	0.03

Adjusted models include the following prespecified variables: age (centered and scaled), sex, ASA, presence of diabetes, presence of hypertension, presence of chronic kidney disease, cardiopulmonary bypass time (centered and scaled), presence of redo sternotomy, acute renal replacement therapy, and presence of tracheostomy. LVEF and LVEDD were centered and scaled. Effect estimates have been exponentiated and are interpreted as percent changes per unit standard deviation. Abbreviations: left ventricle ejection fraction (LVEF), left ventricle end-diastolic diameter (LVEDD).

Across all sensitivity analyses, adjustment for body surface area produced results consistent in direction and magnitude (Supplemental Table 1).

## Discussion

In this study, we evaluated the impact of LV size, a historically overlooked echocardiographic factor in the literature, both alone and in combination with LVEF on outcomes of inotrope therapy, inotrope duration, and ICU LOS in cardiac surgical patients. Lower LVEF was associated with the use of inotrope therapy and greater number of hours of inotrope administration after cardiac surgery. Similarly, dilated LVEDD was associated with the initiation of inotrope therapy; however, dilation of the LVEDD alone was not associated with a prolongation of inotrope duration. This study also demonstrated an association with ICU LOS, such that increases in LVEDD were generally associated with a reduction in the length of ICU stay; however, this association was modified by the preoperative LVEF.

Prior studies have demonstrated that patients with low LVEF have higher mortality rates and higher rates of perioperative complications when compared to patients with normal LVEF [10, 13, 14]. This is logical, considering that these patients experience pathological cardiac remodeling and are susceptible to complications arising from heart failure. This may include lethal arrhythmias [15] and global end-organ malperfusion, although the mechanism may be more complex than simply chronic myocardial hypoperfusion. There may be microvascular dysfunction that contributes to, or potentially better explains, these poor outcomes [16]. However, these studies do not consider the impact of LVEDD or the interplay between LVEDD and LVEF. Our results show that independent of LVEF, a dilated LVEDD is associated with the initiation of inotrope therapy after cardiac surgery.

As previously described by Parrillo et al., LV size is directly proportional to stroke volume and cardiac output, even at a constant LVEF [17]. Patients with a dilated LVEDD and preserved myocardial tissue may have more augmentable stroke volume when compared with cohorts with a normal LVEDD, which could lead to an improvement in cardiac output with the initiation of inotropes. LV dilation in dilated cardiomyopathy may initially serve as a compensatory mechanism to support end-organ perfusion, acting as a protective adaptation to reduced contractility [18]. Endurance athletes undergo cardiac remodeling and plasticity, characterized by an increase in left ventricular end-diastolic diameter (LVEDD). Chronic exercise-induced volume loading is thought to be the mechanism driving an increase in LV diameter. The cardiac output of endurance athletes, therefore, increases significantly with maximal exercise, given the elevated stroke volume [19]. Exercise-induced cardiac remodeling, considered a physiological adaptation, may be challenging to differentiate from pathological dilated cardiomyopathy using echocardiography alone. However, both patient phenotypes may initially benefit from a high absolute stroke volume due to the dilated LV cavity [20]. These basic echocardiographic findings have also led to a more thorough examination of all the cardiac chambers with echocardiography, along with an attempt to better understand not just the impact on systolic function but also diastolic function [21]. Although patients in our study with a dilated LVEDD were more likely to be placed on inotropes, they did not require prolonged inotrope therapy compared to their counterparts with a normal LVEDD. This may be due to the preserved stroke volume typically associated with a dilated LV cavity. It is postulated that this could be due to a preserved stroke volume from their compensatory LV dilation. Increased LVEDD may enhance responsiveness to inotropes by allowing for greater augmentation of stroke volume. Responsiveness to inotropic therapy is quite complex and variable, likely reflecting the net interaction of multiple factors, including LV size, pressure and volume loading conditions, and the contractile state of the heart [22]. Historically, early studies using motion-mode (M-mode) echocardiography, which is a one-dimensional imaging technique used to measure and track motion over time and provides high temporal resolution, useful for precise measurements of moving structures, were conducted to understand myocardial sensitivity to inotropic therapy and overall contractility in response to these therapies [23]. Similarly, a dilated LV cavity can facilitate inotrope weaning, as stroke volume may remain adequate even at a constant ejection fraction. This may explain why inotrope therapy was more frequently initiated in the setting of LV dilation but was not associated with prolonged duration of use. In terms of our secondary outcome of ICU LOS, it is unsurprising that our data showed that patients with both low EF and dilated LV size had longer ICU length of stay, as these are most likely seen in end-stage heart failure patients. Interestingly, among those with preserved LVEF, patients with a dilated LVEDD had shorter ICU LOS, which again may be due to the ability of these patients to augment stroke volume. The dynamic interplay between LVEDD and LVEF is crucial to understanding the complex cardiopulmonary physiology of patients after cardiac surgery.

This study is not without limitations. Data was obtained from a single institution and practices for weaning from inotropes may not be generalizable to other critical care units. At our institution, we utilize a protocol for weaning inotropes based on cardiac index values by both thermodilution and the Fick equation, with a goal cardiac index > 2.2 L/min/m^2^; however, in practice there may be variability in adherence to this protocol where patients may be weaned faster or slower based on individual patient factors and clinician discretion. Despite the rationale for utilizing pre-operative TTE data for baseline echocardiographic characteristics as described in the methods section, there may be patients in whom clinical status changed from the time of their pre-operative TTE to the date of surgery. Another limitation of this study is the lack of immediate postoperative LVEF and LVEDD measurements obtained from intraoperative echocardiography following separation from cardiopulmonary bypass. It is not the current practice within our program to routinely measure immediate postoperative ejection fraction or LVEDD using transesophageal echocardiography following separation from bypass. The predominantly male cohort from a single academic medical center may further limit the generalizability of our results. This study is also limited by its retrospective nature, which is subject to residual confounding and cannot demonstrate evidence of causality, as well as by the inherent limitations of utilizing the electronic medical record. Despite these limitations, this study represents a large, robust dataset that provides important insights into the associations between LVEDD and LVEF on outcomes of patients after cardiac surgery.

## Conclusion

The current study demonstrates an association wherein both dilated LVEDD and low LVEF patients are more likely to be initiated on inotropes, and those with low LVEF are also more likely to have a prolonged duration of inotrope therapy. Despite a historical research focus solely on LVEF, this study demonstrates that in addition to LVEF, preoperative LVEDD is an important echocardiographic variable that may aid in identifying patients at risk for adverse outcomes after undergoing cardiac surgery.

## Notes

**Supplemental Table 1:** Sensitivity analysis of inotrope therapy and ICU length of stay outcomes after adjusting for body surface area

	**Effect Estimate (95% CI)**	**P Value**
Presence of ICU Inotrope Therapy		
LVEF	0.45 (0.41 to 0.50)	<0.001
LVEDD	1.19 (1.07 to 1.32)	0.001

Hours of ICU Inotrope Therapy		
LVEF	0.75 (0.68 to 0.82)	<0.001
LVEDD	0.97 (0.88 to 1.06)	0.47

ICU Hours		
LVEF	0.89 (0.87 to 0.92)	<0.001
LVEDD	0.97 (0.94 to 1.00)	0.03
LVEF * LV	0.98 (0.95 to 1.00)	0.03

Each model was adjusted for body surface area (centered and scaled), as well as the pre-specified covariates listed in the Methods. Abbreviations: left ventricle ejection fraction (LVEF), left ventricle end-diastolic diameter (LVEDD).
